# Supplementary material for: Cloud BioLinux: pre-configured and on-demand bioinformatics computing for the genomics community
Source: BMC Bioinformatics. 2012 Mar 19;13:42. doi: 10.1186/1471-2105-13-42 (PMC3372431; doi:10.1186/1471-2105-13-42)
Supplement: Additional file 1 — Supplementary 1 Cloud BioLinux software documentation in the form of a mini, self-contained website. Users need to download and uncompress the .zip file, and open through a web browser the "index.html" file available on the main directory. (ZIP 1823 kb). [file 1471-2105-13-42-S1.ZIP › Cloud-BioLinux-Package-Documentation/docs/njplot.html]

Bio-Linux Software Documentation Pages

Back to search form

## njplot

|  |  |
| --- | --- |
| Name | njplot |
| Description | **njplot** allows phylogenetic trees read from Newick formatted files to be displayed, re-rooted, saved, and plotted to PostScript (or PICT for the Macintosh) files.   Input trees can be with/without branch lengths, with/without bootstrap values, rooted or unrooted. O�nly binary trees are accepted.  **References:**  PerriXre, G. and Gouy, M. (1996) WWW-Query: An o�n-line retrieval system for biological sequence banks. Biochimie, 78, 364-369. |
| Homepage | http://pbil.univ-lyon1.fr/software/njplot.html |
| Remote Documentation | ftp://pbil.univ-lyon1.fr/pub/mol\_phylogeny/njplot/njplot.help |
